# Understanding biomaterial-tissue interface quality: combined *in vitro* evaluation

**DOI:** 10.1080/14686996.2017.1348872

**Published:** 2017-07-31

**Authors:** Michael Gasik

**Affiliations:** ^a^ School of Chemical Engineering, Aalto University Foundation, Finland

**Keywords:** Biomaterial, simulation, testing, *in vitro*, biomechanics, dynamics, implant, 30 Bio-inspired and biomedical materials, 211 Scaffold / Tissue engineering / Drug delivery, Biomaterials, Implants, Testing

## Abstract

One of the greatest challenges in the development of new medical products and devices remains in providing maximal patient safety, efficacy and suitability for the purpose. A ‘good quality’ of the tissue-implant interface is one of the most critical factors for the success of the implant integration. In this paper this challenge is being discussed from the point of view of basic stimuli combination to experimental testing. The focus is in particular on bacterial effects on tissue-implant interaction (for different materials). The demonstration of the experimental evaluation of the tissue-implant interface is for dental abutment with mucosal contact. This shows that testing of the interface quality could be the most relevant in controlled conditions, which mimic as possible the clinical applications, but consider variables being under the control of the evaluator.

## Graphical abstract

**Figure:**
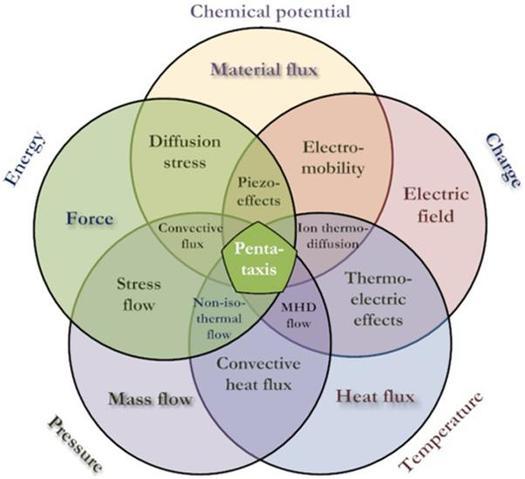

## 1. Introduction

One of the greatest challenges in the development of new medical products and devices remains in providing maximal patient safety, efficacy and suitability for the purpose. It is generally understood that ‘good quality’ of the tissue-implant interface is one of the most critical factors for the success of the implant integration and restoration of the functionality of that tissue or organ in question. It is, however, not easy to define terminologically what is ‘good’ and what is ‘quality’, even for implants of the same kind, like orthopedic or dental cases. What can be more precisely defined is the type and grade of biomaterial used in that implant, biomaterial composition, surface state, chemistry, etc., as these parameters are now being measured with great precision and can be also documented to allow post-marketing follow-up. Many types of biomaterials are presently available for use in different implants [1,2]: metallic alloys, ceramics, composites, polymers are all used whether with or without live cells, medical substances or other additions like antifouling or antibacterial factors. There is also a growing trend of use of different scaffolds in tissue engineering applications with the purpose of support and promotion of correct tissue formation.

Any ‘excellent’ developed medical device might cause complications and adverse effects. It is not possible to foresee how and when medical treatment will be carried out for a specific patient, and in many cases the only way is to rely on the experience and intuition of the doctor. To approach this challenge, *in vivo* tests are commonly considered to be a ‘gold standard’ to determine how an implant would act in such conditions.

However, the great burden of variables and uneven conditions makes most of the animal tests essentially useless, raising the costs and slowing the introduction of new solutions. Most of the *in vivo* tests that ‘well went’ fail in human clinical trials (success rates of only 2%–15% have been reported [3]). The decisions from *in vivo* tests are often made on limited, weakly controlled limited populations of subjects with a high scatter. In Europe the Directive 2010/63/EC requires medical device manufacturers to move towards the ‘3R’ (Reduction, Refinement, Replacement) approach for humane science. This drives manufacturers, developers and clinicians to strengthen the application of alternative *in vitro* and *in silico* methods to obtain the maximal information from the intended product before undergoing clinical trials [4,5].

Most of existing *in vitro* methods still rely on very limited conditions, are fragmented and are thus not sufficiently translatable to patient circumstances. As an example, one may recall biocompatibility evaluation using ISO 10993 procedures. It is notable that the *Biomaterials* journal editorial warns that any manuscript referring to such procedures will be rejected, as these biocompatibility evaluations have no scientific reasoning and are done purely for regulatory purposes.

Hence there is still a lack of reliable, reproducible and reasonable alternative *in vitro* methods, capable of producing most of the biomaterial and implant readouts, which would have a direct clinical relevance, whether or not they are correlated with animal *in vivo* tests. To achieve ‘good quality’ of the tissue-implant interface one need to know: 1) how to assess the quality of this interface, 2) how to translate this knowledge into quantified readouts and endpoints, and 3) how to ensure that this quality outcome would be sufficiently stable for a variety of patient conditions (anatomy, physiology, presence of diseases, sex, age, lifestyle, expectation of comfort and restoration). Even the first challenge alone is not easy – use of massive *in vivo* tests is prohibitive, both ethically and also in time and costs. For evaluation of biomaterials the hostile-like *in vitro* environments (closest to the respective clinical conditions) are desirable with control of chemical, biological, mechanical and other parameters [1,2]. This is of importance in high-throughput screening (HTS), usually implemented as a large number of smaller test cases (e.g. culture wells) generating a massive array of data, being post-processed and visualized for the purpose of finding correlations and trends (known as computational discovery).

In orthopedic and dental materials [1,6] as well as advanced therapy medicinal products (ATMPs) containing stem cells, this approach is difficult to realize, as the implantation site cannot be reasonably squeezed to microliter volumes, without any natural-mimicking stimuli. It is anticipated that in medical devices another approach should be introduced as ‘high-output screening’ (HOS). In contrast to HTS, it aims to maximize the amount of consistent data and information from the minimal number of tests or specimens. This could provide great assistance for medical device producers, researchers and medical doctors as it will minimize clinical tests, shorten time to market and improve many lives, without compromising patient safety.

In view of the above, here we discuss some critical features of such an approach. First, we consider some general fundamental aspects and then see some peculiarities of biomaterials and bacteria interaction, as the prevention of primary infection is one of utmost importance. Second, we will look in more detail at the case of a dental implant – one of the most commonly used worldwide. Third, we will show an example of the application of the new simple biomechanical test to analyze the tissue-implant quality.

## 2. Some fundamental aspects of biomaterials and tissue interactions

When the implant is deployed into a patient, it faces an environment with a lot of variables, which are unknown, uncontrolled and varying in an unpredictable manner (hematoma, inflammation, bioenvironmental attack, proteins and species absorption, cells’ competitive attachment, bacterial invasion, etc.). Any one of these processes is sufficiently complex on its own and not very well known. Insufficient and scattered data of clinical practice do not allow conclusions on how the device behaves over time and, in particular, during the first days and weeks post-procedure. Information concerning e.g. stress state, displacement monitoring, biochemical environment changes, etc. is fundamental to promote high durability of the implantable device, prevention of infection and finally to provide comfort and reliability to the patient.

**Figure 1.**
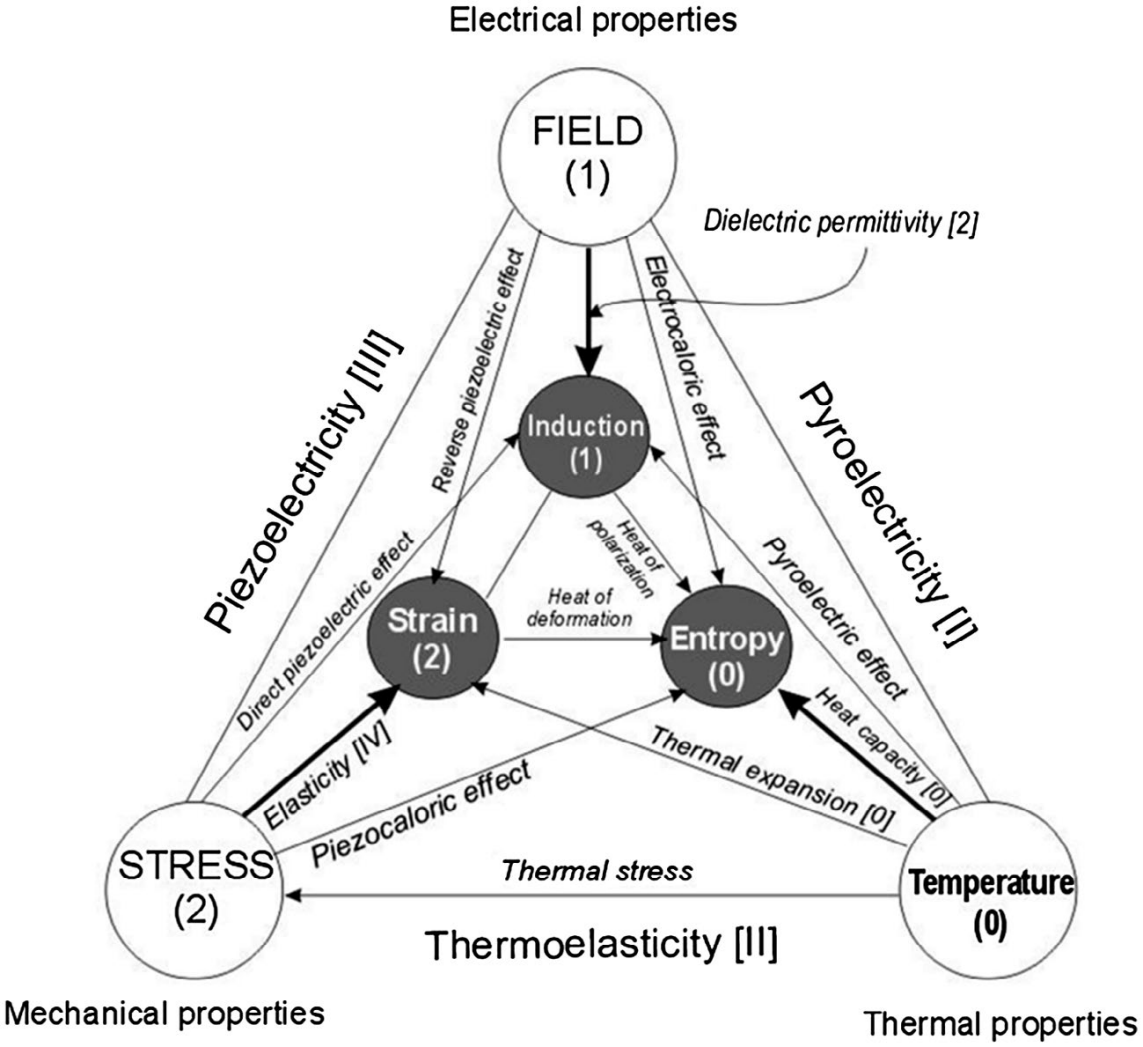
Principal connections and interactions between the physical stimuli and materials parameters. Numbers in brackets indicate respective tensor rank.

**Figure 2.**
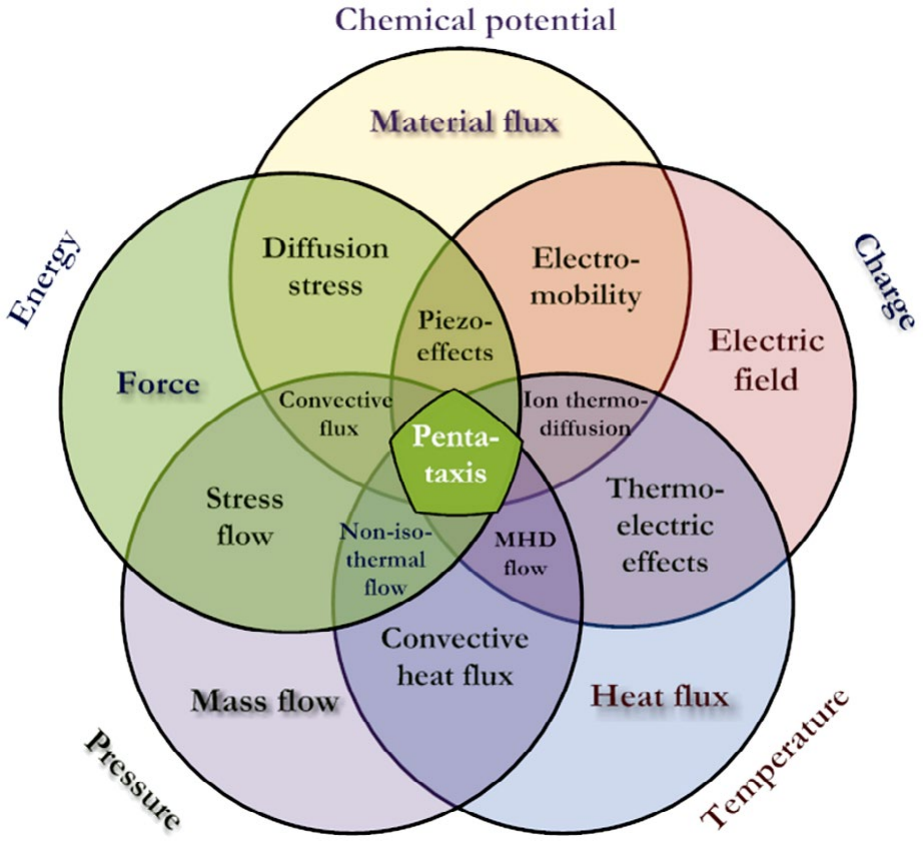
The philosophy of pentataxis concept (MHD, magnetohydrodynamics).

Similarly to the basic system of fundamental units (SI) one may consider that development of the tissue (cell evolution) is being driven by some basic forces. In cell research the term ‘*taxis*’ (movement) is widely used to describe for instance cell migration on a biomaterial surface due to chemical composition differences (*chemotaxis*) [7] or due to differences in stiffness (*durotaxis*) [8]. In physics, the relations between basic factors like temperature, pressure, electrical field strength are well known, and the connections between them are known as different physical phenomena (Figure 1). For biomaterials and living tissues we suggest the ‘*pentataxis*’ concept (Figure 2), in which main five factors are responsible for the majority of all effects practically observed: temperature, pressure, energy, electric charge and chemical potential of the species. The ‘Big Five’ driving forces for these are respective gradients or differentials of the factors (Table 1), which in their interactions cause a large variety of effects acting on cells, membranes, extracellular matrix and of course on biomaterials. For example, electric current caused by an electric field (potential difference) would lead to charge separation across the membrane, which in turn may cause movement of charged ions. This will trigger respective reactions with molecules, proteins and eventually response of cells inherited into tissue formation or transformation. Every stage of this process might be very complex and also affected by other external factors and driving forces. It might be that the full picture would be impossible to describe in a consistent set of equations so some surrogate models are needed to link stimuli and reactions for practical applications.

**Table 1.** Fundamental stimuli and *pentataxis* forces with the respective phenomena (simplified).

Gradients of stimuli:	Fundamental acting stimuli				
	Temperature (T)	Pressure (P)	Charge (Q)	Energy (U)	Chemical potential (μ)
∇T→heat flux **q**	Conductivity, radiation	Thermal flow	Thermoelectric current	Thermal stress	Thermal diffusion
∇P→fluid flow **V**	Convection	Viscosity	Baro-electric current	Fluid stress	Baro-diffusion
∇Q→electric field **E**	Peltier heat flux	Streaming potential	Electrical conductivity	Polarization stress	Electromobility, electro-osmosis
∇U→ force **F**	Dissipation heat	Fluid pumping	Piezoelectricity	Stiffness	Stress diffusion
∇μ→ species flux **J**	Convective heat	Swelling, shrinking	Charge separation (e.g. membranes)	Concentration stress	Diffusivity

Let us consider as an example the behavior of implant and tissue from a biomechanical point of view. Investigation of mechanical properties of biological systems is a very complicated objective and requires specific knowledge of every part of this system [1,2,9]. It is known that biomechanical properties are critical for biological processes, and these properties regulate the signals sent to the cells [9–11]. Due to the variety of parameters involved and the obvious limitations of *in vivo* studies, computational mechanobiology is often applied to determine the quantitative rules that govern the effects of mechanical loading on tissue differentiation, growth, adaptation and maintenance, by trial-and-error.

Even in the case of biomechanical behavior of the natural (bone, cartilage) or artificial (relevant implants), without inclusion of cellular signals, biochemical pathways and external environment, it is very difficult to obtain an engineering model capable of prediction of the behavior of such materials, needed for their proper design [9]. Not so many models take into account the surrounding tissue properties, and none considers interface area properties, which in most of the cases remain unknown. Mechanical instability at interfaces (tearing, excessive shear, movement, rupture, etc.), whether or not complicated by possible biofilm formation, is one on the main reasons of the implant loosening and its subsequent failure.

From the point of view of *pentataxis*, one may consider this part of the problem by trying to identify the links and correlations between the stimulus (= acting driving force, primary or secondary) and tissue reactions. The latter are case (and maybe patient) dependent, but it should be possible to express tissue reaction in some objective (gene expressions, biomarkers, bacterial analysis) and subjective (pain, discomfort) metrics. To understand how for instance bacterial attachment may affect tissue-implant interface quality and how it could be tested or simulated, one needs to look in more detail at the bacterial impact on different typical biomaterials.

## 3. Biomaterials and bacteria interactions

### 3.1. Biofilm problem in modern implantology

As any surgery or medical intervention, an implantation might have complications, of which those associated with acute and delayed prosthetic joint infections (PJI) remain a high concern. PJI are a devastating complication after arthroplasty and are associated with substantial patient morbidity [12]. It compromises patients’ quality of life, causing pain and immobility and generally requiring two-stage additional operations entailing bone, muscle and soft tissue loss. Extended hospital stays and operations expose the patients to multi-resistant pathogens, putting them at greater risk of secondary complications or even death (more than 25% of revisions are attributed to these infections [13,14]). The increased prevalence of obesity, diabetes and other co-morbidities are some of the reasons for this increase. They are expected to increase in the USA alone by 673% for total hip and 174% for total knee arthroplasties by 2030, with the annual revision costs to hospitals increased from $320 million to $566 million during 2001–2009, projected to exceed $1,620 million by 2020 [14]. Many ‘aseptic loosening’ cases known in the past were misidentified or not properly reported, more than 70% being caused by bacteria [15–17].

Hospital and health-care facilities are peculiar environments in which dangerous antibiotic-resistant pathogens can live and evolve. Hospital-based pathogens show continuous dynamic change, and this influences their distribution through the body over time and their pathogenicity [18]. With emerging numbers of antibiotics-resistant strains, the efficacy of antibiotic treatment might be low and further suppressed due to formation of biofilm. Overcoming the multi-drug resistance (MDR) of several bacteria is still a major global challenge. The reported death rates due to MRSA (methicillin-resistant *Staphylococcus aureus*) infections are 20,000–40,000 mortalities annually in the USA and ~25,000 in the EU [19]. Most of the microorganisms causing infections are present in the host flora (including those on skin) and cannot be fully avoided. Common biofilm-related medical device infections (including dental cases) are due to the Gram-negative *Pseudomonas aeruginosa*, *P. fluorescens* or *Escherichia coli* or to the Gram-positive *Staphylococcus epidermidis*, *S. aureus* or enterococci. Staphylococci are associated with metallic and polymer orthopedic implants, and an antibiotic treatment application is a common practice [20–22]. Vertebral infections caused by *S. aureus* are usually associated with chills, weight loss, photophobia and drainage from a wound or incision. Antibacterial treatment of infected implants is difficult and usually requires their removal [21]. The presence of an implant decreases the minimal infecting dose of *S. aureus* 100,000-fold for causing a permanent abscess [20]. Pathogens entering the bloodstream increase the risk of e.g. cardiovascular and pulmonary diseases, hinder control in diabetes and contribute to peri-implant infections leading to implant loss.

Besides bacterial ones, fungal species are also known to cause similar problems. For instance, in the case of vascular catheter-related infections, the most commonly isolated microbial species are Gram-positive *S. epidermidis* and *S. aureus*, and the fungus *Candida albicans* [23]. Fungal infections (mycoses) are significantly increasing in incidence throughout the world as a result of modern medical practice (expanding use of immunosuppressive therapies, broad-spectrum antibiotics, central venous access devices, organ and stem cell transplantation, as well as presence of foreign bodies (implants) [24]). Sepsis due to fungal infection increased more than 200% in the USA alone between 1979 and 2000 [25].

### 3.2. Biofilm formation at the biomaterial-tissue interface

Why and how is biofilm formed on any surface? Once inserted into the host site, the implant biomaterial soon (in micro- to milliseconds) becomes coated with components of the surrounding body fluids (conditioning film). Depending on the body site, the surrounding fluids can be saliva, urine, tear fluid, tissue fluid, blood, etc. The conditioning film will consist mostly of adsorbed proteins such as fibronectin and fibrinogen, which can act as receptors for bacterial attachment [26]. However, the manner in which conditioning films influence microbial attachment remains unclear. Physicochemical surface properties of the biomaterial and bacteria cell surface are believed to play a major role in this conditioning and adhesion process [22]. Different local physical and chemical factors (electric potential, surface energy, chemical activity), local environment (pH, ionic strength, oxygen tension), surface topography, porosity, tortuosity, hydrophobicity, instant and longer-term microfluidics are all affecting the adhesion.

Mechanical properties are very important for selection of treatment or dispersal of biofilm organisms due to a body fluid’s flow and associated motion of the surfaces in question [27]. In general, the mechanical properties of a biofilm determine the deformation of a biofilm due to an applied force, such as shear and more complex deformations [28]. Biofilms can be mechanically challenged during growth, in the oral cavity during fluid flow arising from powered tooth-brushing and tongue movement, from pulsatile blood flow in intravascular catheters, or from the movement of tissues, fluid and biomaterial components in an orthopedic joint prosthesis [28].

It is well known that rough materials accumulate more biofilms and dental plaque and expose patients to the risk of diseases at neighboring sites. This is a key aspect in implantology, because most implants available on the market are designed to be rough and grooved, in order to improve primary stability, healing of mineralized and soft tissues and maintenance of tissue integration around the implants over time [29]. When rough surfaces are exposed to the proper environment, biofilm formation is rather fast. The clinical roughness threshold for biofilm formation in the oral cavity is R_a_ ~0.2 μm [30]; below this threshold, for R_a_ values within the microscale, there is no significant improvement in inhibiting bacterial adhesion [31]. In contrast, at the nanoscale, rough and geometrically determined surface morphology has been shown to produce antifouling properties. At this scale, interaction of the bacteria with the surface remains limited to the surface of physical protrusions [32]. However, at the nanometer scale bacterial adhesion does not always follow the roughness of the device surface but is also strongly related to other variables such as the total amount and properties of adsorbed proteins [33]. Furthermore, bacteria adhesion can be also affected by surface structure in terms of short-range van der Waals interactions and surface energy [34].

The main *in vitro* method of assessment of bacterial interaction with a biomaterial is direct culturing of bacteria seeded on the surface, with subsequent analysis of results with microscopy, biomarkers, laser flow cytometry and other techniques [29–31,35]. Every analytical method has its own benefits and drawbacks, and none could be recommended for all occasions.

The behavior of biofilm on flat surfaces is different from ones residing inside very rough surfaces or in porous bodies (coatings or similar) [20,35]. Analysis of bacterial adhesion and biofilm formation inside porous coatings is very challenging and might not be feasible with some standard techniques [36]. Besides the ‘optimal’ implant antibacterial function (not considering specific drug-carrying surfaces), the requirements for osteoblast fixation and mechanical and biomechanical constraints set additional challenges for implant developers, because in many cases almost incompatible demands have to be met.

Biofilms on implant surfaces are not only the origin of infection, although the last-mentioned is definitely the major medical concern. Bacterial activity can destroy biomaterial and its surface, cause loosening due to weakening of the biomaterial-tissue interface, promote biomaterial corrosion [37] and affect its frictional features. For example, for dental biofilms on titanium it was shown that pH of the medium in which biofilms grow decreased in the presence of microorganisms, probably due to the release of acidic substances, which reduced the corrosion resistance of titanium [38]. Thus the presence of lactic acid-producing bacteria such as *S. mutans* can increase the corrosion of Ti-based systems used for oral rehabilitation. Also, it was noted in the same study that a fresh titanium area is exposed to an environment that contains corrosive substances such as those resulting from a microbial metabolism. A wear-corrosion process that takes place during sliding of titanium parts in a corrosive environment can thus be a cause of failure in dental implant-supported systems.

#### 3.2.1. Metallic biomaterials

Several metallic alloys are being widely employed in orthopedic and dental areas, the major ones being titanium alloys, stainless steels, cobalt-chromium, noble metals and some shape memory alloys [39]. Of these, titanium-based materials have been widely used because of their mechanical strength, corrosion resistance and biocompatibility [40–42]. Despite high rates of clinical success, biofilm-associated infections have emerged there as a leading failure mechanism, caused mainly by staphylococci, streptococci, *Pseudomonas* spp. and coliform bacteria. Although increased hydrophilicity of the biomaterial surface is believed to be beneficial in minimizing the biofilm formation risks, combined quantitative analyses between the actual implant parameters and bacterial development are still fragmented. For example, streptococcus species are able to colonize titanium even after hydrophilic or hydrophobic surface modifications [43]; therefore, the colonization degree of titanium implants was overridden by surface roughness and irregularities rather than charge.

Orthopedic titanium implant surfaces exhibit different roughness types, surface treatment and other features, usually designed to promote osseointegration and mechanical contact between the implant and host tissues [44,45]. All titanium (in general all metallic) implants might be roughly divided into those with a highly porous surface (normally coatings such as vacuum plasma sprayed [VPS] titanium) and those without it (polished, sandblasted, etched or otherwise treated). Furthermore, any of this type may be additionally coated with an external layer (hydroxyapatite, bioactive glass, etc.), and it is generally well known that such modification would critically affect surface roughness, porosity, wetting ability and consequently cell and bacterial adhesion to the implant. Together with biomechanical factors, these set the main boundary conditions for bone in-growth and osteointegration. Roughened titanium surfaces enhance the focal contacts for cellular adherence, and they guide cytoskeletal assembly and membrane receptor organization [46–48].

Analysis of the simultaneous effect of bacteria and cells has been performed *in vitro* in one study [49], where not only roughness and presence of TiO_2_ on various titanium surfaces were compared, but also the effect of porosity, topology, manufacturing methods, resulting wetting angle and cultivation time were considered. The formation and colonization of *S. aureus* and *S. epidermidis* at 1, 2 and 3 days, as well as human endothelial and human osteogenic cell proliferation up to 27 days on these surfaces were correlated with gene expression (CD31, von Willebrandt factor, alkaline phosphatase and collagen I). For example, a threshold of ~47% in porosity was found to indicate that highly porous titanium materials would exhibit an intrinsic risk of biofilm formation despite attempts to make them more hydrophilic or more smooth. On the other hand, low porosity alone does not guarantee that biofilm formation is less risky, but in that case the effect of hydrophilic treatment, the TiO_2_ presence and adjustment of other topologic parameters are more pronounced. These experimental data indicate the possibility of decreasing the biofilm formation by 80%–90% for flat substrates versus untreated VPS porous titanium and by 65%–95% for other porous titanium coatings [49]. It was also shown that optimized surfaces would lead to 10%–50% enhanced cell proliferation and differentiation versus reference porous VPS titanium coatings. This presents an opportunity to manufacture implants with intrinsic reduced infection risk, yet without the additional use of antibacterial substances. Thus hydrophilicity of an implant alone is not at all sufficient to ‘guarantee’ positive results, as it might be achieved in different ways.

#### 3.2.3. Polymeric biomaterials

The types of polymers applied in orthopedic and dental load-bearing practice are usually limited to polyethylene (PE) with different density and molecular weight, polymethylmethacrylate (PMMA) as for bone cement, and fluorinated polymers such as PTFE and polyetheretherketone (PEEK) families [50–53]. In cartilage repair, polylactic acid isomer (PLA, PLLA, PLDA) based materials and composites are also employed due to their biodegradable properties [54,55].

As for metallic surfaces, local topography and chemistry of polymers are very important factors in bacterial adhesion. The interaction of bacteria with two surfaces of identical chemistry but differing topography can result in significantly different densities of adherent bacteria *in vitro* [56,57]. Usually roughening a surface increases the available surface area for colonization and might generate more turbulent fluid flow, but on the other hand it might also change dynamic hydrophilic nature of the surface. It is generally understood that fluid circulation close to an implant is nearly laminar (although not always), but local flow disturbances are naturally possible. In comparison to metals, many polymers have additional variations in crystallinity, phase structures, presence of residuals (stabilizers, modifiers, etc.), as well as composite constituents (fibers of different composition and orientation). These constituents also respond differently to humidity (swelling), environmental factors and naturally to the proteins in question [58,59]. For example, residual non-polymerized monomers in dental resins, such as triethyleneglycol- and 2-hydroxyethyl-methacrylates, leak from the materials, diffusing into the oral cavity surroundings [60]. Due to toxicity of these monomers to gingival fibroblasts, the latter actually benefit from the early biofilm formation by *Streptococcus mitis* to protect themselves from the acute reaction. Eventually, at later stages, biofilm in any case becomes contributive to various disease states like gingivitis, root surface caries and periodontitis.

Cross-linked polyethylene is still used in combination with metal or ceramic implantable devices. However, massive polymer surface degradation has been strongly related to the appearance of osteolytic process, where the presence of debris is one of the most common key risk factors. It has been demonstrated that particle-derived implant side effects are directly related to their size, shape and concentration. The first host reaction to these particles is macrophage activation; then, also the cytokines release results are increased, leading to severe tissue response such as bone resorption [61].

#### 3.2.3. Ceramic biomaterials

As with all other materials, all bioceramics might be colonized by bacteria, and all are eventually capable of making biofilms [29]. In general, comparison of zirconia and other ceramic devices for orthopedic and dental applications has revealed that these materials had an intrinsic ability to reduce or at least delay biofilm formation. For example, yttrium-stabilized zirconia for dental applications has been evaluated for their ability to form biofilms *in vitro* and *in vivo* for *Streptococcus mutans*, *S. sanguis*, *Actinomyces viscosus*, *A. naeslundii* and *Porphyromonas gingivalis* [62]. The surface state of the zirconia ceramics was made compatible to commercial abutments (R_a_ ~0.10–0.30 μm). *S. mutans* was observed to attach more easily to zirconia than to titanium control, but no differences were seen for various zirconia preparation methods. Similar results were obtained in human follow-up analysis performed in zirconium oxide discs. The lower bacteria adhesion ratio for zirconia in comparison with titanium disks was probably due to the superﬁcial structure of zirconium oxide [63].

Generally, no porous and high-performance mechanical ceramic materials seem to have intrinsic antibacterial properties. For instance, no differences in biofilm formation were observed clinically between polished and glazed yttrium-stabilized tetragonal zirconia polycrystalline ceramic for dental prosthetic reconstructions. The small amount of bacteria recovered from polished surfaces was probably due to the superior surface smoothness, confirming what has previously been debated about the importance of surface roughness for first bacteria adhesion [64]. The use of ceramic materials such as zirconia might be favored in place of metals or polymers because of numerous evidences *in vitro* and *in vivo* of their intrinsic ability to reduce bacteria adhesion. It might be speculated that for all types of bioinert ceramics bacterial attachment mainly proceeds through the pioneering selective proteins adhesion, for which fine acting forces (van der Waals, electrostatic, etc.) differ from metallic surfaces due to the nature of conductivity and potentials formed in body fluid environments [29].

For some biomaterials one option is grafting/doping other metal ions capable of reducing biofilm formation. Ions like silver are known to have an oligodynamic effect (toxic effect on living organisms) on microorganisms [65–67].

Antibiotics (despite the growing concerns of drug resistance) application remains one of the main actions against biofilm-related infections. Traditional protocols might be rather ineffective, which relates to the lower efficacy of antibiotics on bacteria residing in biofilms. In dental practice, infection rates as high as 30% were seen despite prophylaxis [68]. It is common knowledge that antibiotics are in general not very capable of destroying biofilms. However, this is not completely true, as it depends on the biofilms and antibiotics type, dose, administration procedure and location, and only certain antibiotics appear to target biofilms effectively.

Therefore, for any type of a biomaterial, there is always a risk of biofilm formation and infection, but the properties of biofilm and its virulent degree are highly individual and cannot be in general approximated by some engineering functions. In this case, the regulative norms prescribe that the ‘worst case’ scenario has to be considered in designing, testing and validating biomaterials for implants.

## 4. Case: dental implants and abutments

### 4.1. Dental implant structure and tissue peculiarities

Dental implant systems should have a firm attachment of the implant to the bone and soft tissue to an abutment, as this is critical for long-term stability and health of the implant system. A good peri-implant (i.e. adjacent to the implant surface) soft tissue attachment protects a dental implant system from bacterial infections, which in the worst case might severely resorb peri-implant soft tissue and adjacent bone. Implant-abutment structure can be obtained by using one- or two-piece implants, but in any case they need different surface structures to get the optimal attachment on both bone and oral mucosa [69]. Gingiva, as a soft tissue covering the jaw bone, is attached to a tooth by junctional epithelium seal. A similar permeable seal can also be formed between a gingiva and an abutment. Thus, as small a gap as possible is desired to reduce the probability of bacterial penetration [70,71]. Gingival properties are difficult to quantify due to its heterogeneous composite structure. Differences between individuals can stem from different parameters such as age, sex, life style choices and inflammations such as gingivitis. For example, gingiva becomes less elastic when aging [71]. The orientation of collagen fibers in connective tissue is important for the structure of a gingiva. The collagen fiber structure of keratinized mucosa provides better attachment for teeth allowing it to endure more mastication frictions and forces. Thus, for a good quality of abutment-tissue interface, keratinized epithelia should be enhanced in gingiva [70].

The essential differences between periodontal (i.e. adjacent to native dental tissue) and peri-implant soft tissues and their attachments in the case of titanium abutments are shown in Table 2. Attachment mechanisms of periodontal and peri-implant soft tissues are rather similar, the biggest differences coming from different surfaces and wound healing processes caused by implantation. Due to implantation, peri-implant tissue resembles a scar tissue. Thus, vascularization is poorer in the 40–50 μm zone from the titanium surface due to the small amount of blood vessels, which can lead to lowered immune response. A hydrophilic surface can enhance vascularization and thus the stability of an abutment [72,73].

**Table 2.** Comparison of periodontal (= adjacent to natural tooth) and peri-implant (= adjacent to implant biomaterial) soft tissues and their attachment (adapted from [72,75]).

	Periodontal soft tissue	Peri-implant soft tissue
Attachment to …	root cementum	directly to implant surface
Supported by …	alveolar bone, periodontal ligament, cementum	basal lamina, hemidesmosomes
Connective tissue collagen bundles are…	perpendicular	parallel
Connective tissue composition has …	60% collagen, 5%–15% fibroblasts	85% collagen, 1%–3% fibroblasts
Vascular plexus blood supply via …	periodontal ligament	missing or insufficient
Wound healing acting for …	no wound healing	initial phase → location of junctional epithelium. Granulation tissue → can result in loosening of abutment

### 4.2. The dental tissue-implant interface formation

**Figure 3.**
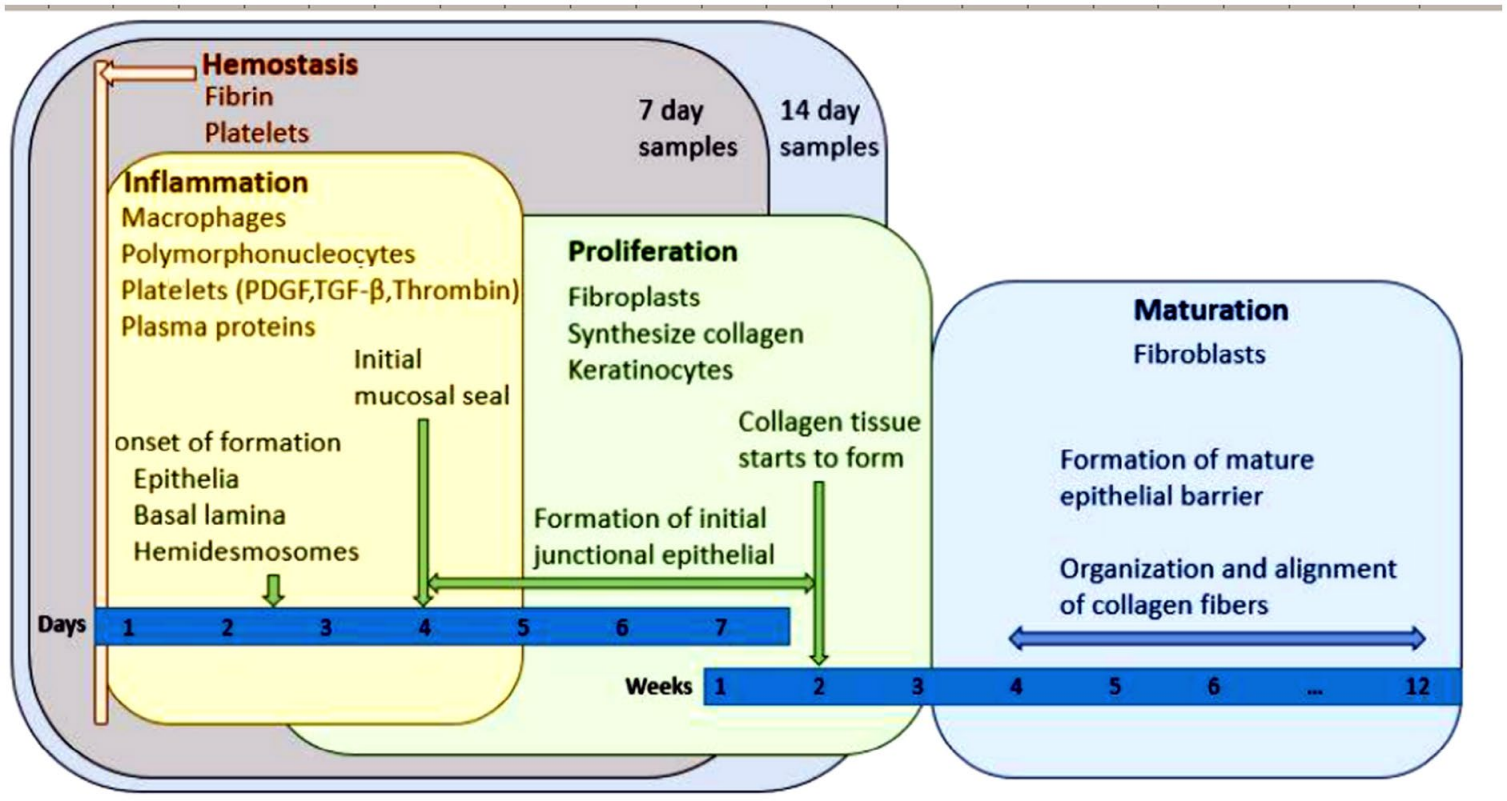
Timeline for healing of an implant and readout areas for 7 and 14 days *in vitro*, adapted from [72].

An abutment attachment is initialized within the first seconds after the implantation of an abutment. First a water layer is placed on an abutment in nanoseconds followed by the second layer attached to it by hydration and steric forces [74]. Acellular salivary biofilm of three layers (phosphoproteins and low- and high-molecular-weight glycoproteins) forms in this layer, depending on pH, flow rate and composition of saliva. It helps to minimize bacterial attachment by reducing interfacial free energy and increasing strains for some bacteria [74]. After the initial biological responses a delayed response follows: cell attachment and proliferation, tissue reactions, which can enhance or prevent healing. Enhancing reactions include contact, connection, growth and differentiation of cells. Decreasing tissue reactions include rejection, encapsulation, resorption, thrombogenesis and ectopic calcification (Figure 3) [72,74]. Here is also marked position for specimens for *in vitro* testing (7 days and 14 days) as it is a reasonable compromise whether or not a ‘good quality’ junction has been formed [72]. Peri-implant tissue healing is a long process [75], but it should be allowed to proceed undisturbed. If an attachment between an abutment and soft tissue is ruptured repeatedly, the height of a gingiva is reduced. This reduction affects mainly the height of the connective tissue, while the height of the epithelial tissue stays the same [76].

### 4.3. Biomechanical effects in dental implants

Dental implant and abutment faces many mechanical and biological factors in an oral environment, and they all affect the attachment to the tissues. Mechanical loads during mastication, grinding and parafunctional clenching directly create mechanical stress in the implants and respectively cause micromotions on the tissue interface [77]. Mastication stresses depend on individual characteristics such as age, sex, bone quality, soft tissue characteristics and used food type [78]. The frequency of the mastication is usually around 1 Hz, which relates to one occlusion of the teeth per second. Force and stresses are non-uniform, causing additional wear due to some parafunctional chewing habits, neuromuscular forces and abrasion of food and antagonists [72,79]. In addition to macroscopic forces, micro-scale forces (at ~1–200 μm scale) are associated with fluid stress, blood and saliva movement, local tissue deformations, etc. Whereas they can be but small, at a single cell scale (~10–30 μm) they could be critical, causing membrane deformations, regulating ion channels and eventually signaling to and between cells for proper direction of the proliferation and evolution. Such forces are difficult to predict and control, but they have a great impact on the new tissue formation and also tissue-implant interface development. The third important contribution associated with macro- and microscopic forces is various biological and biochemical factors. These include, for example, pumping effect of fluid, which affect local pH, salinity, delivery of species and removal of metabolic products, etc.

For any biomaterial of the dental implant, bacteria are exploiting all three cases to attach to the surfaces: (1) an immediate, earliest competitive attachment after implantation, (2) accumulation in to a biofilm during use, and (3) follow-up penetration (infiltration) of bacteria through the permeable epithelial junction seal. The most critical one is the first, as in the case that bacteria would occupy the surface there would be no space for cells and mucosal tissue to attach well. This creates a continuous source of infection and will lead to implant failure. As this process happens during the first hours and days, it is possible to evaluate *in vitro* up to 2 weeks’ time (Figure 3). From the above analysis, one may conclude that one of the options for carrying out such tests, and assessing whether or not one implant or material would be better than another one, would be mimicking the conditions of the implant location. This would have to combine all the forces and fluid flows (at least to the controllable and reasonable extent) and possibly use ‘worst case’ bacterial loading to see the effect of potentially formed biofilm on the adhesion of the tissue.

## 5. Biomaterials enhanced simulation test (BEST) *in vitro*

A prerequisite in the evaluation of any biomaterial is the creation of a suitable and relevant testing environment, whether or not the material specimen contains live cells. In the case of added cells, most testing conditions (temperature, time, atmosphere, media) are being dictated or fixed due to cell culture requirements and may not be too much varied. In a wider analysis, one may also be interested in the behavior of a biomaterial beyond the limits of its application to assess critical factors such as variations of humidity, temperature or sterilization method on materials property [80]. For example, if a material is steam-sterilized, does it change porosity or elastic modulus, and, if yes, how much?

For the dental implant case shown above, the most important practical endpoints are the improvement of the (1) tissue-biomaterial adherence, (2) resistance to potential bacterial contamination and biofilm formation, and (3) absence of potentially hazardous earlier adverse effects. For example, a biomaterial loaded with antibiotics or silver would definitely minimize the risk of biofilm formation, but at the same time it might inhibit or even prevent cell growth and attachment. This nevertheless might be still acceptable in specific patients (HIV carriers, the immunodepressed) as other potential risks are much larger.

**Figure 4.**
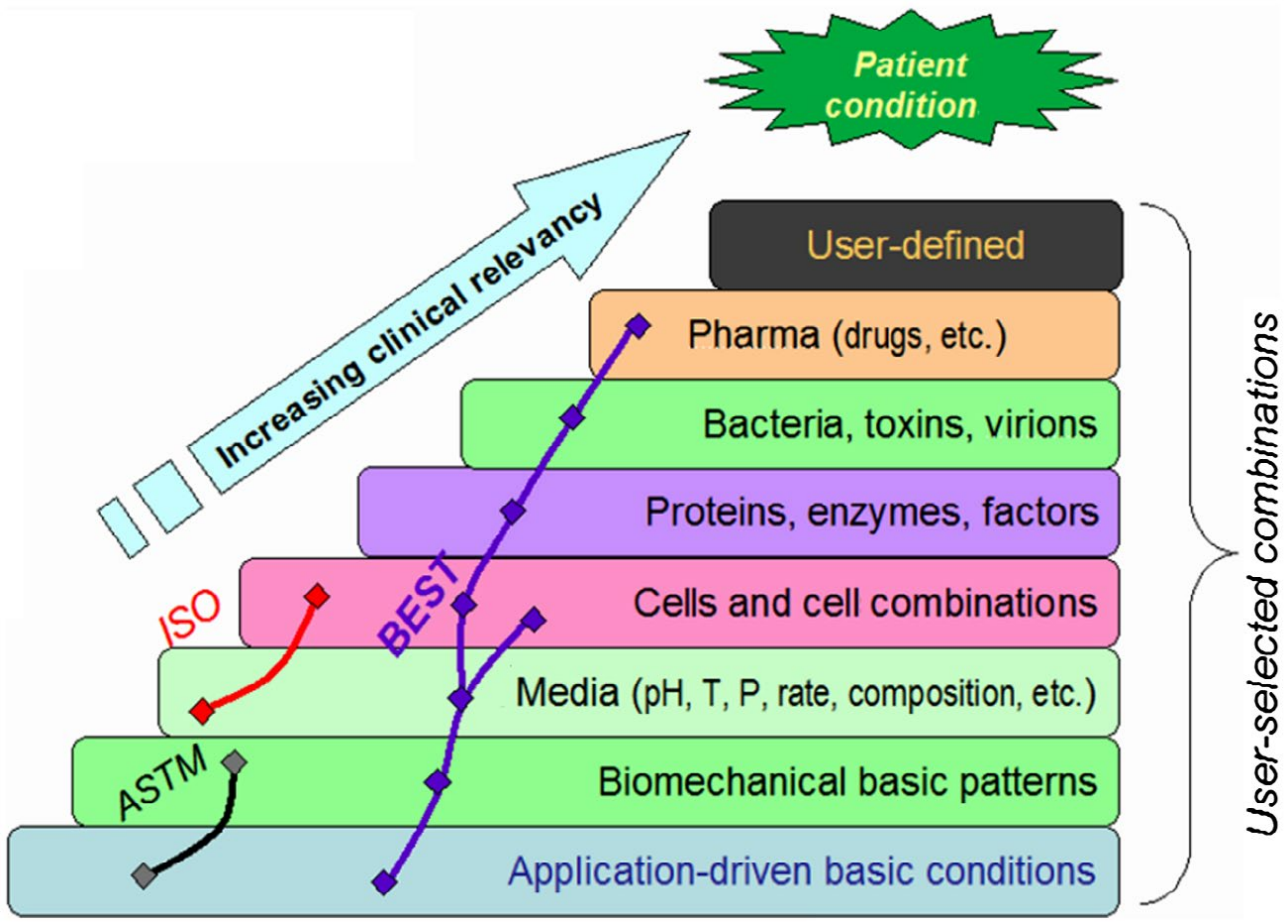
The concept of the biomaterials enhanced simulation testing [80–82].

With these features and boundary conditions in mind, a set of protocols was designed and implemented as BEST, biomaterials enhanced simulation testing [80–82], for the purpose of evaluation of biomaterials under proper biomechanical conditions with additions of the user-selected variables potentially of interest (Figure 4).

**Figure 5.**
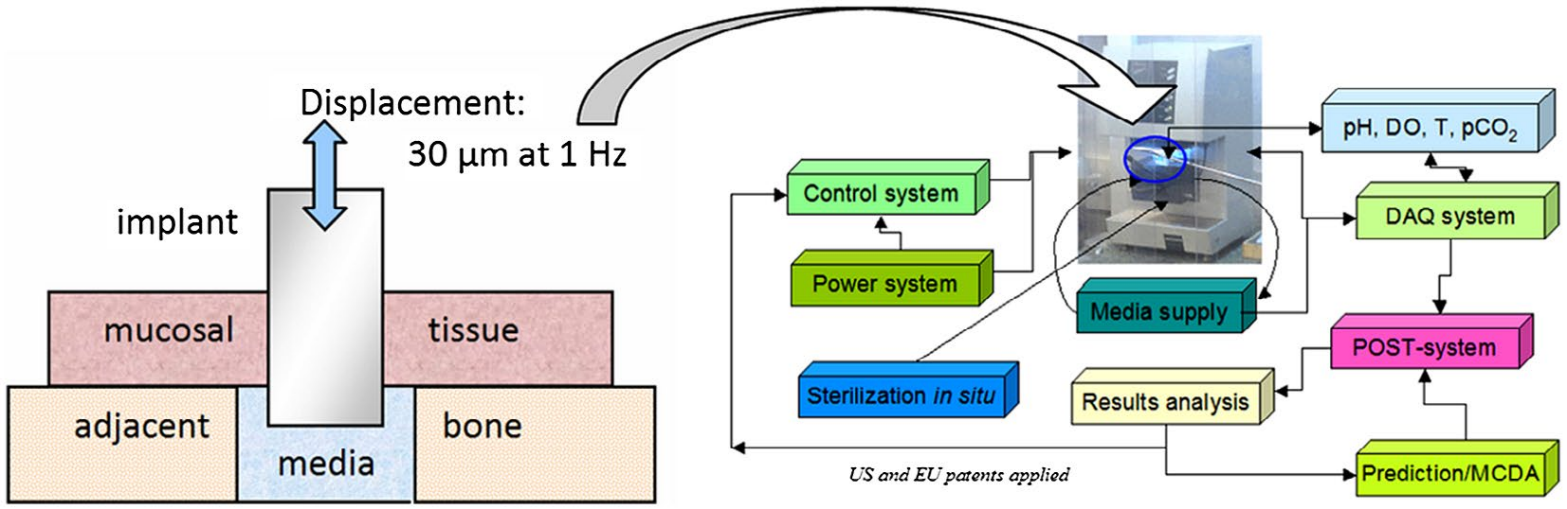
The dental abutment test concept (based on [72]) for the BEST platform (DO, dissolved oxygen; DAQ, data acquisition; POST, post-processing data treatment; pCO_2_, CO_2_ partial pressure; MCDA, multi-criteria decision aiding).

**Figure 6.**
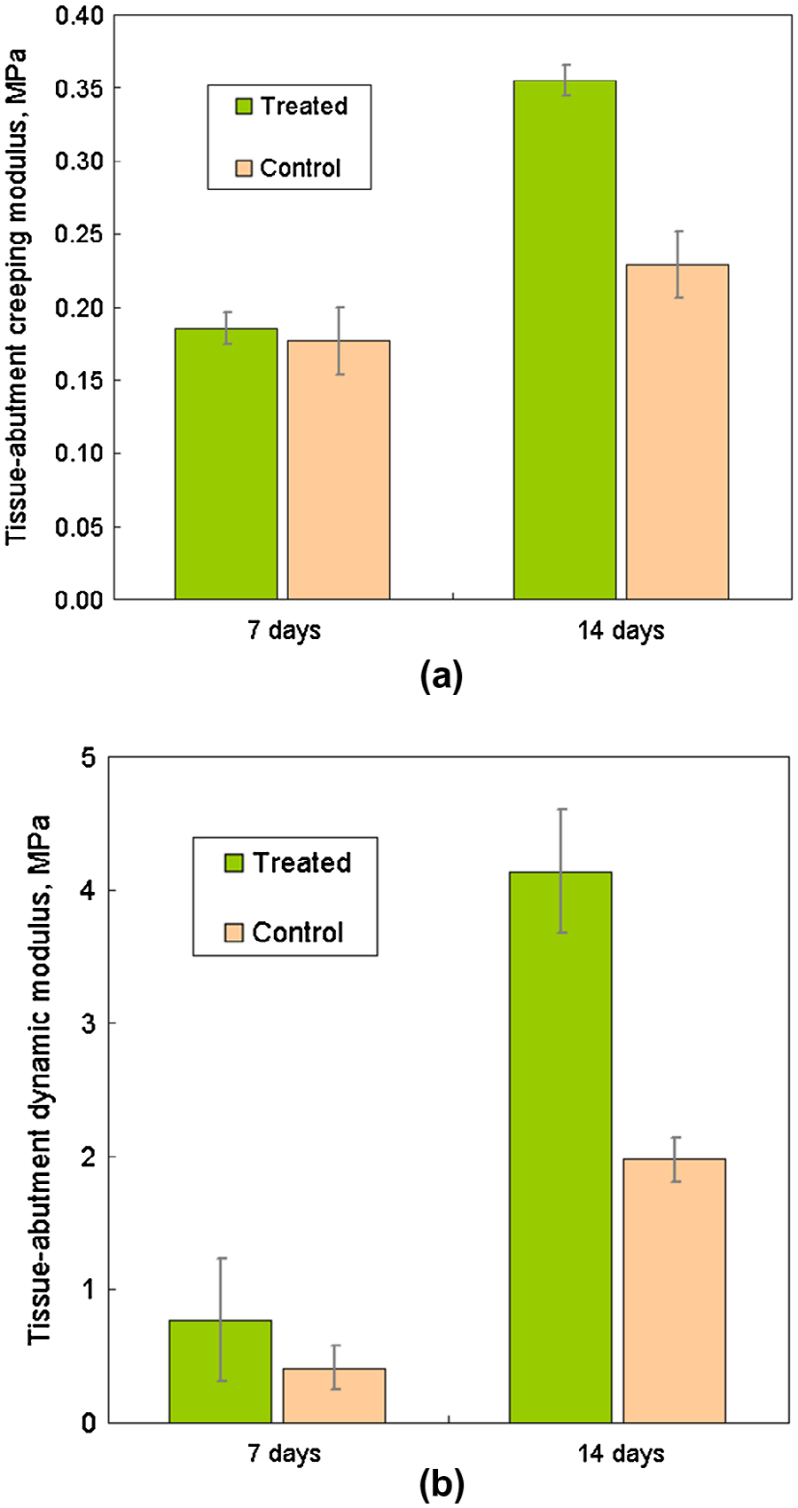
An example of mucosal tissue-abutment interface quality measurement with BEST (Figure 5), expressed as modulus under: a) pseudo-static loading part (‘creeping’); and b) dynamic loading (1 Hz, 30 μm deformation amplitude).

The essential difference of BEST vs. existing standards is that the latter use mainly oversimplified *in vitro* conditions which are not too close to clinical reality. One option of the layout of the BEST platform and the logical links between the hardware and software parts are shown in Figure 5 together with the simple setup for evaluation of the tissue-abutment biomaterial interface quality. Here instead of dynamic load control (force), displacement control (strain amplitude) is implemented of 30 μm and 1 Hz frequency. These are the most common parameters dental abutment and the implant are facing in reality [82–84]. The abutment pins can be cultivated either separately and then tested, or cultivated under dynamic conditions if so required [72,85]. An example of these simple measurements is shown in Figure 6 for abutments treated for repelling bacteria (lower biofilm formation risk) vs. control of the same material. Here formal ‘modulus’ is used to assess how firmly the abutment pin is bound to the tissue – the higher the value, the better is the attachment. It can be seen that after 7 days of cultivation (Figure 3) differences in pseudo-static modulus are very small, but in dynamics differences are already detectable. After 14 days of cultivation, both static and dynamic parameters are shown to gain significant differences, being 70%–100% higher for treated biomaterial. It might be assumed that dynamic loading in physiological conditions could be considered as the earliest marker of the biomaterial-tissue interaction in this case, and it could be the measure for HOS when several biomaterial options are to be compared quickly before more detailed evaluation.

For HOS purposes many other parameters for the interface quality assessment can be also measured (directly or as a post-processing of the data) just in one experiment: dynamic (shear modulus, bending modulus, loss tangent, cyclic decay, etc.) and pseudo-static (displacement change, creeping compliance, history-dependent viscoelastic properties, etc.). Variation of geometry (e.g. hole diameter or abutment size) gives an additional leverage of the wider stresses and strain ranges. Also mucosal tissue alone (without underlying bone) can be cultured and tested on artificial supports if bone-mucosa adhesion is not a subject of investigation.

When bacteria are added, or pH, temperature, media composition are changed, new data sets can be compared and the decision made for the selection of the best biomaterial solution [82]. This drastically reduces the time and effort required for *in vivo* or clinical tests, as the solutions not showing a ‘good quality’ would be unlikely to succeed in any extended deeper tests.

## 6. Conclusions

The tissue-implant interface quality is a complex feature including a lot of contributions and is treated differently even for the same material-tissue combination used in different anatomic locations (e.g. zirconia in orthopedics and in abutments). The dynamics of the interface development gives more challenges in its characterization and produces more scattered results. This makes results comparison between different studies very challenging, if not impossible.

New solutions in advanced and more consistent evaluations for biomaterials are needed to cope with costs, community demands, quality/risk control and regulatory requirements. This can be improved with combined (mechanical, fluidic, biological) tests and models with multi-purpose protocols to secure patient safety by certifying biomaterial in hostile-like conditions. In this review only parts of the big problem were considered, such as bacterial interaction with biomaterials and its effect on the interface quality. An example for dental implant and abutment testing, using the BEST platform, was presented, and similar protocols can be tailored for rather complex clinical conditions, as shown e.g. for articular cartilage repair [86,87].

Thus it is possible to mimic and control the most significant conditions *in vitro* aiming to provide high-output screening, to evaluate the effect of different parameters on tissue-implant interface quality and to select lead biomaterials candidates for further application.

## Disclosure statement

No potential conflict of interest was reported by the author.

## Disclaimer

In accordance with Taylor & Francis policy and my ethical obligation as a researcher, I am reporting that I have a business interest in Seqvera Ltd that may be affected by the research reported in the enclosed paper. The results presented here are in no way considered as a promotion or encouragement for that company’s business operations, nor impacting on scientific or technical merits or presented research outcomes.

## Funding

This work was partially supported by the Finnish Agency for Innovation (Tekes) under Grant 40190/13.
